# First-Principles Study of Mo Segregation in MoNi(111): Effects of Chemisorbed Atomic Oxygen

**DOI:** 10.3390/ma9010005

**Published:** 2015-12-26

**Authors:** Yanlin Yu, Wei Xiao, Jianwei Wang, Ligen Wang

**Affiliations:** 1 General Research Institute for Nonferrous Metals, Beijing 100088, China; yuyanlin_121@163.com (Y.Y.); wxiao@ustb.edu.cn (W.X.); jswjw@sina.com (J.W.); 2 School of Materials Science and Engineering, University of Science and Technology Beijing, Beijing 100083, China; 3 Power Environmental Energy Research Institute, Covina, CA 91722, USA

**Keywords:** density-functional theory calculation, surface segregation, hydrogen evolution electrode, oxygen chemisorptions, water electrolysis

## Abstract

Segregation at metal alloy surfaces is an important issue because many electrochemical and catalytic properties are directly correlated to the surface composition. We have performed density functional theory calculations for Mo segregation in MoNi(111) in the presence of chemisorbed atomic oxygen. In particular, the coverage dependence and possible adsorption-induced segregation phenomena are addressed by investigating segregation energies of the Mo atom in MoNi(111). The theoretical calculated results show that the Mo atom prefers to be embedded in the bulk for the clean MoNi(111), while it segregates to the top-most layer when the oxygen coverage is thicker than 1/9 monolayer (ML). Furthermore, we analyze the densities of states for the clean and oxygen-chemisorbed MoNi(111), and see a strong covalent bonding between Mo d-band states and O p-states. The present study provides valuable insight for exploring practical applications of Ni-based alloys as hydrogen evolution electrodes.

## 1. Introduction

Adding a second metal into a pure metal catalyst can provide a great opportunity to tailor the properties of the catalyst [1,2,3,4]. Therefore, bimetallic systems have attracted considerable attention due to their importance in basic science and industry [5,6,7,8]. Bimetallic systems are much more complicated than pure metal catalysts since one element may segregate to the surface and lead to surface composition enrichment/depletion compared to the bulk. The adsorbate-induced surface segregation of metallic alloys under the reaction conditions and, thus, the changes in local atomic composition and surface structure have been predicted and demonstrated to occur for a number of bimetallic systems [9,10]. So for a given bimetallic configuration which exhibits a desired property, it is crucial to know whether the particular configuration is stable under the operating environment for a specific application.

Many theoretical and experimental studies have been performed to investigate the surface segregation for various bimetallic systems [11,12,13,14,15,16,17,18,19,20,21,22,23,24,25,26,27,28,29]. The recent experimental and theoretical works focusing on the description of bimetallic and ternary, extended and nanosized, alloy surfaces under reactive gas phase environments were reviewed by Zafeiratos, *et al.* [19] and by Guesmi [20]. On the theory side, Guesmi and co-workers had performed extensive first-principles calculations to investigate the transition metal segregation behaviors for the gold-based bimetallic systems in the presence of various gas adsorptions [11,12,21,22,23]. Specifically, the authors found that segregation of Pd on PdAu(111) is oxygen coverage–dependent and that Pd atoms tend to be in the bulk for the clean surface while they segregate to the surface in the presence of more than 1/3 ML of oxygen [12]. The surface phase diagrams were investigated within the first-principles atomistic thermodynamics framework by addressing the effect of the bulk alloy and the gas phase reservoir [13,24,25,26]. The first-principles-based cluster expansion technique [27] had also been employed to model the surface ordering and segregation of alloys in a reactive environment [28,29]. This approach allows us to explore enormous different atomic configurations. Although the latter two methodologies are very useful for studying the alloy surface ordering and segregation in the presence of adsorbates, the direct first-principles calculations [11,12,21,22,23] are certainly able to predict the segregation tendency of alloy systems. The advantage of the direct first-principles calculation method is that it is accurate and computationally more affordable, while its drawback is that it cannot predict what equilibrium phases and structures will form under given conditions.

Among the bimetallic systems, nickel-based alloys are especially interesting because of their applications in a number of catalytic reactions including the hydrodeoxygenation of esters [30], methanation reaction [31], and propane reforming [32]. In the context of hydrogen evolution reaction (HER), recent experiments showed that adding some Mo element into nickel-based electrodes could improve the electro-catalytic activity [33]. Previously, the calculations without any gas adsorption and the experiments in a ultra high vacuum (UHV) chamber showed that it is difficult for Mo atoms to segregate to the surfaces in NiMo alloys [34,35,36]. However, several previous experiments found that Mo atoms segregate to the NiMo alloy surfaces and induce an enrichment of the surface Mo composition under the reaction conditions [17,34]. The composition and structure of Ni-2 at % Mo(100), Ni-2 at % Mo(110) and Ni-6 at % Mo(110) surfaces were studied by Auger electron spectroscopy (AES) and low-energy electron diffraction (LEED) [34]. The authors obtained the molybdenum pre-enriched surfaces by annealing the samples in purified hydrogen. We also experimentally investigated the Mo surface segregation by energy-dispersive spectroscopy (EDS) in the NiMoCo electrolysis electrode materials [17]. In our experiments, the Ni at 20.8 wt % and Mo at 1.6 wt % Co foam electrodes were prepared by the electro-deposition method and decarbonized at atmospheric conditions to remove the residual carbon due to the polyurethane foams, followed by a reduction process in hydrogen atmosphere for removing the surface oxides. This Mo surface enrichment (40 wt % Mo for the surface *vs.* the nominal 20.8 wt % Mo in the bulk) could be because of the presence of chemisorbed atomic oxygen during the decarbonation of the MoNi electrodes [17]. Obviously, detailed theoretical calculations are desirable and helpful to understand the experimental observation.

In this paper, we investigate the effects of chemisorbed atomic oxygen on the segregation behavior of the Mo element in MoNi(111) by performing first-principles calculations. The (111) surface is likely the dominant facet for the NiMo electrolysis electrodes since, among various Ni surfaces, Ni(111) is the most stable one. Based on the previous studies [22,35] we believe that, if we consider other orientations, the segregation behaviors will not change. This is because the surface segregation has a weak dependence on the orientation [22]. Mo segregation on NiMo(111) and NiMo(100) in vacuum has been calculated and the results show that the Mo atom has the same segregation tendency for the two surfaces [35,36]. In the work by Sansa, *et al.* [22], the authors found that the stronger adsorption energies of M impurities on the (100) alloy surfaces compared to the (111) surfaces do not induce a better segregation toward the (100) facet. The authors argued that the adsorption anisotropy is mostly generated by the matrix metal Au and slightly depends on the chemical nature of M. Inconsistent with previous studies [35,36], our calculations show that the surface segregation of the Mo element does not occur in the vacuum, namely for the clean MoNi(111) it is energetically unfavorable for Mo atoms to segregate onto the surface. In the presence of chemisorbed atomic oxygen, the Mo element is found to segregate to the alloy surface when the coverage is thicker than 1/9 ML. By carefully analyzing the densities of states, we see that the preference of Mo atoms to be embedded in the Ni bulk is mainly governed by surface energy considerations and atomic size effects. We also find a strong covalent bonding between Mo d-states and O p-states. The remainder of the paper is organized as follows. In Section 2, the theoretical methods and computational details are described. Section 3 presents the calculated results of segregation energies, and electronic structure analyses. A brief summary is given in Section 4.

## 2. Computational Methods

The first-principles calculations were performed within a density functional theory using the Vienna Ab-initio Simulation Package (VASP) [37,38,39]. The electron-ion interaction was described using the projector augmented wave method (PAW) [40,41] and the exchange correlation potential using the Perdew–Burke–Ernzerhof (PBE) functional method [42]. The energy cutoff for the plane wave basis set was 450 eV for all the calculated systems. Spin polarization was taken into account in the calculations and the Methfessel and Paxton [43] was employed to determine electron occupancies with a smearing parameter σ of 0.14 eV. The convergence criteria for the electronic self-consistent iteration and the ionic relaxation loop were set to 10^−5^ eV and 0.02 eV/A, respectively.

**Figure 1:**
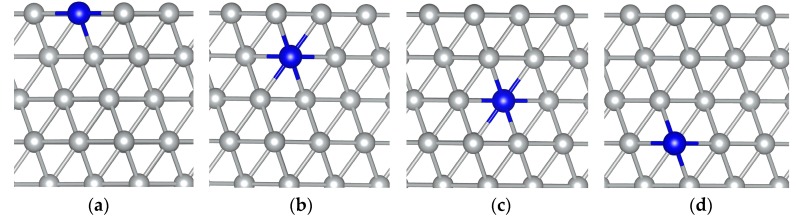
MoNi(111) alloy systems showing one Mo monomer substituting one Ni atom in the (**a**) first; (**b**) second; (**c**) third; and (**d**) fourth nickel layer. Only the four top layers are shown. Gray and blue balls represent Ni and Mo atoms, respectively.

To simulate metallic surfaces, a slab supercell was employed. All the calculations presented in this work were based on slabs of 54 atoms, containing six atomic layers representing a 3 × 3 supercell, separated by 15 A of vacuum space. MoNi(111) alloy systems corresponding to the substitution of one Ni atom by one Mo atom in the first, second, third or fourth nickel layer are presented in Figure 1, respectively. The Mo atom plays a role as a prober to determine whether it prefers to stay on the surface or in the bulk. As pointed out above, the drawback of this method is that it cannot predict the equilibrium phases and structures. However, investigation of the equilibrium phases and structures under certain given conditions is beyond the scope of the present study. Chemisorbed atomic oxygen was located on only one side of the slab. The influence of the resulting electric dipole on the computed energy values was estimated to be very small according to standard methods [44], and had thus been neglected. The atoms in the top four layers and the chemisorbed atomic oxygen were allowed to relax, while the atoms in the bottom two layers were fixed at the bulk geometry positions. Brillouin zone integrations were performed using Monkhorst–Pack grids [45] of 4 × 4 × 1 for slab calculations. The gas-phase oxygen molecule was simulated through a large supercell with dimensions of 12 × 12 × 12 A^3^.

In order to characterize the segregation behavior of the Mo atom in nickel, the segregation energy (*E_segr_*) was defined as the energy difference between the states with the Mo atom located at the upper surface layer and in the bulk. According to this definition, the segregation energies were calculated according to the following equation:

(1) $$E_{segr}=E_{MoNi\left(Mo,x-layer\right)}-E_{MoNi\left(Mo,4th-layer\right)}$$

where *E_MoNi(Mo,x-layer_*_)_ represents the total energy of the MoNi alloy system with the Mo atom located in the upper x nickel layers (*x* = 1, 2 or 3), and *E_MoNi_*_(*Mo,*4*th-layer*)_ represents the total energy of the MoNi alloy system with the Mo atom located in the fourth nickel layer, which corresponds to the presence of the Mo atom in the “bulk” nickel matrix. For the oxygen adsorption cases, *E_MoNi_*_(*Mo,x-layer*)_ and *E_MoNi_*_(*Mo,*4*th-layer*)_ are the total energies of the slabs with O atoms adsorbed on the top layer and the Mo atom located at the corresponding atomic layer. According to our calculations, a chemisorbed atomic oxygen prefers to occupy a three-fold fcc hollow site on MoNi(111) and Ni(111). For higher oxygen coverage cases (such as 2/9, 3/9, and 4/9 ML oxygen coverages corresponding to the adsorption of two, three and four oxygen atoms, respectively), we considered all possible oxygen adsorption configurations and calculated the segregation behavior by using the most stable adsorption configurations.

## 3. Results and Discussion

**Figure 2:**
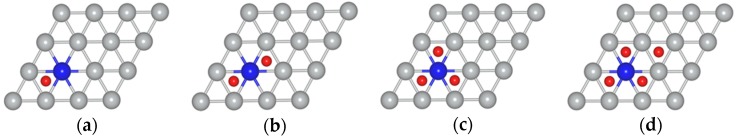
Top views of the most stable adsorption configurations for the oxygen coverage of (**a**) 1/9; (**b**) 2/9; (**c**) 3/9 and (**d**) 4/9 with Mo located at the top-most atomic layer of the slab. Only the top-most metal atomic layer and adsorbed O atoms are shown. Gray, blue and red balls represent Ni, Mo and O, respectively.

**Figure 3:**
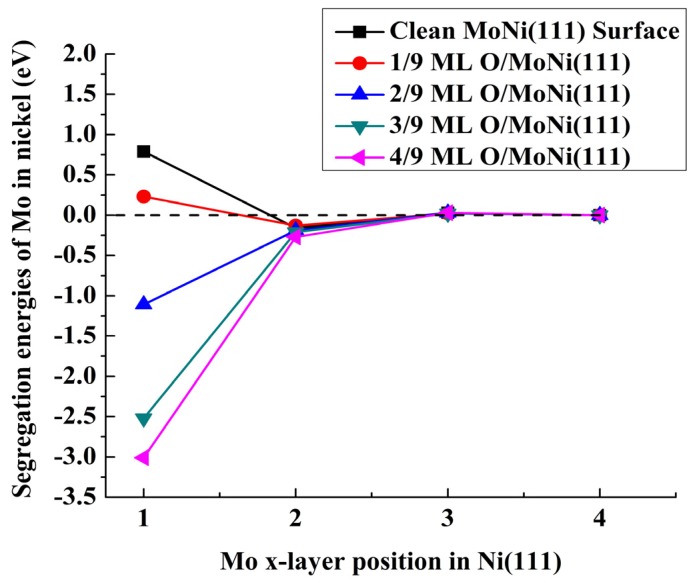
Evolution of the segregation energies (eV) of Mo atom from the nickel “bulk” (fourth layer) to upper layers toward the surface in the presence of different atomic oxygen coverage.

We have done an exhaustive and extensive search on the lowest-energy O adsorption patterns (or arrangements) for each oxygen coverage with the Mo atom located at various atomic layers. The most stable O adsorption configurations for Mo located in the top-most atomic layer are shown in Figure 2. For the 1/9 ML oxygen coverage case, the O atom prefers to occupy a fcc site near the surface Mo atom. The two O atoms occupy a fcc site and an hcp site next to the Mo atom, respectively, for the oxygen coverage of 2/9 ML It is less stable by 0.16 eV for the two O atoms to occupy the two fcc sites. For the higher oxygen coverage cases (such as 3/9 ML and 4/9 ML) O atoms first occupy the fcc sites next to the surface Mo atom and then those fcc sites far away from the Mo atom. We find that the subsurface Mo atom prefers to stay away from the adsorbed O atoms; for instance, when the Mo atom is located at the second atomic layer, O atoms occupying the fcc sites far away from the top position of the Mo atom are energetically more favorable. When the Mo atom is located at the third or lower atomic layer, it imposes a negligible effect on the O adsorption, as we can see from Figure 3, that the segregation energies do not rely on the oxygen coverage.

The calculated segregation energies for clean and oxygen- adsorbed surfaces are given in Table 1 and also plotted in Figure 3. In absence of adsorbed gas, *i.e.*, under vacuum conditions, the segregation energy for Mo located at the top-most layer is positive and has a value of 0.79 eV. This indicates that Mo does not segregate to the top-most surface layer. We can attribute this behavior to the smaller surface energy of nickel compared to that of molybdenum (experimental values are 2.45 eV/atom for Ni and 3.00 eV/atom for Mo [46]). Interestingly, the Mo atom prefers to occupy a site below the top-most surface layer. This oscillatory phenomenon is quite common in alloying systems [47,48,49,50,51]. That the Mo atoms located at the second layer are energetically favorable can be understood by two facts: (1) since it is not on the top-most layer it can avoid the higher surface energy of Mo; and (2) by locating at the second layer, the system can largely release the elastic energy due to the atomic size mismatch.

At the oxygen coverage of 1/9 ML, although the segregation energy drops to 0.23 eV from the value of 0.79 eV for the clean surface, the Mo atom still prefers to locate inside of the bulk. With increasing the oxygen coverage to 2/9 ML or thicker, the Mo atom becomes more stable in the top-most layer than in the lower layer and in the bulk. This result indicates that the presence of chemisorbed oxygen on the surface of a MoNi alloy electrode, such as during the decarbonation of the electrode, may cause the surface to be Mo-rich, *i.e.,* having a concentration higher than the nominal concentration in the alloy. This explains our recent experimental observation that the electrode surface is Mo-rich [17]. We can understand the O adsorption-driven segregation of Mo as follows. There are two factors that favor Mo to segregate onto the top-most layer. The first one is that segregation of the Mo atom onto the top-most layer helps to release the elastic energy caused by the size mismatch. Another factor is that the Mo-O bond is stronger than the Ni-O bond (from the chemical rubber company (CRC) handbook [52], the bond strengths for diatomic Mo-O and Ni-O molecules are 145.1 kcal/mol and 93.6 kcal/mol at 298 K, respectively). There exists a factor that is against the segregation of Mo onto the first surface layer. This unfavorable factor is that the Mo surfaces have a larger surface energy than the corresponding Ni surfaces. The final segregation behavior is determined by the competition between the two favorable factors and the unfavorable factor.

**Table 1:** Calculated segregation energies for MoNi alloy configurations with different adsorbed atomic oxygen coverage. The reported segregation energies of molybdenum in O-MoNi(111) systems are calculated by considering the most energetically stable adsorbed oxygen configurations.

Position of the Mo Atom	Atomic Oxygen Sub-Monolayer on MoNi(111) Alloy				
	0	1/9 ML	2/9 ML	3/9 ML	4/9 ML
	*E_tot_* (eV)				
First layer	0.79	0.23	−1.11	−2.52	−3.01
Second layer	−0.16	−0.13	−0.19	−0.21	−0.27
Third layer	0.03	0.02	0.03	0.02	0.03
Fourth layer	0	0	0	0	0

**Figure 4:**
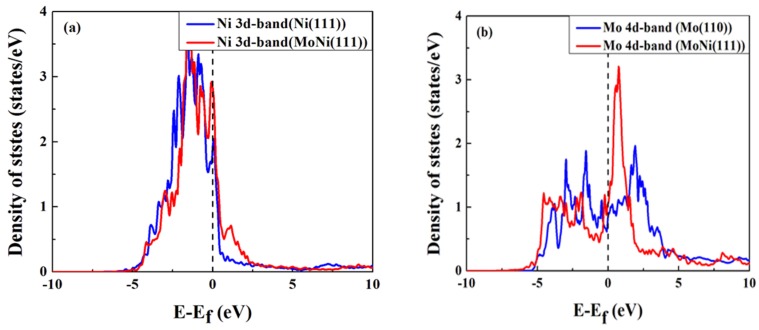
Calculated density of states (DOS) of the Mo and Ni atoms in the alloy and pure metal surfaces. (**a**) d-band DOS of Ni atom in the top-most atomic layer for the alloy and pure Ni surfaces; (**b**) d-band DOS of Mo atom in the top-most atomic layer for the alloy and pure Mo surfaces.

The d-band densities of states (DOS) for Mo and its nearest neighboring nickel atoms in the top-most surface layer with and without oxygen adsorption were calculated. Figure 4 shows the d-band DOS for both molybdenum and nickel atoms in the MoNi alloy compared to their corresponding DOS in pure metals. The d-band DOS for Ni(111) and Mo(110) are presented because they are the most stable surfaces for the metals. Compared to the pure metal surface atom, the nickel d-band DOS center is slightly shifted up towards the Fermi level in the alloy case (Figure 4a), while the molybdenum d-band DOS center is shifted towards the lower energy region, away from the Fermi level (Figure 4b). According to the d-band center model developed by Hammer, *et al.* [53], these d-band center shifts closely correlate to oxygen adsorption–induced Mo surface segregation as we have observed above. There exists an additional peak at the higher edge of the d-band DOS for the alloy case (Figure 4a). This peak indicates the formation of a covalent Ni-Mo bond [12]. From Figure 4b we can see that the Mo d-band not only shifts to the lower energy region, it also becomes narrower than in the pure metal, and the empty states are fewer. This means that the Mo atom gains some electrons from its neighboring nickel atoms, which is consistent with the d-band DOS of Ni shifting up and having more empty states in the alloy case (Figure 4a).

**Figure 5:**
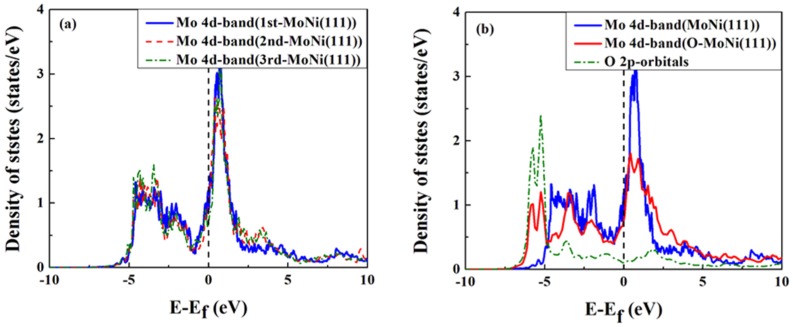
(**a**) d-band DOS for the Mo atom located in the first, second and third MoNi(111) layers in absence of oxygen; (**b**) d-band DOS for the Mo atom located in the top-most atomic layer with and without oxygen adsorption and oxygen p-band DOS.

The d-band DOS for the Mo atom located at the top-most surface layer, the second layer and the third layer without oxygen adsorption are presented in Figure 5a. From Figure 5a, we see the shape and position of the Mo d-band DOS do not significantly change no matter which layer the Mo atom is located at. This indicates that the preference of Mo atoms to locate in the Ni bulk is mainly governed by surface energy considerations and atomic size effects (2.39 A for Mo and 2.22 A for Ni [54]) [12]. Figure 5b shows that the d-band DOS of Mo in MoNi(111) interacting with oxygen are drastically modified compared to those for the clean surface (*i.e.*, without oxygen adsorption). The d-band DOS for the oxygen adsorption case are largely widened. The narrower DOS for the surface atoms are easy to understand, given they have a smaller coordination number relative to the bulk atoms. For the adsorption cases, it can be considered that as the adsorbate increases the coordination number for the surface atoms, it therefore broadens the DOS [55]. From Figure 5b, we can see that the broadening of the Mo d-band DOS causes some d-states to move to the lower energy region upon O adsorption. This effect lowers the system energy and leads to better stability of the system. Furthermore, we also see that the O-Mo anti-bonding states, located above the Fermi level, are largely unoccupied and that the O p-states and Mo d-states have a large overlap, thus allowing a strong covalent bonding and hybridization interaction between molybdenum and the oxygen atom.

## 4. Conclusions

We have performed density functional theory calculations to investigate the effects of chemisorbed atomic oxygen on the segregation behavior of the Mo element in MoNi(111). In particular, the coverage dependence and possible adsorption-induced segregation phenomena are addressed by calculating the segregation energies of the Mo atom in the upper layers of MoNi(111). The theoretical calculated results show that the Mo atom prefers to be embedded in the bulk in the clean MoNi(111), while it segregates to the top-most layer when the oxygen coverage is thicker than 1/9 ML For the clean MoNi(111) we see that the d-band center of Ni atoms surrounding the Mo atom shifts up to the Fermi energy and the Mo d-band becomes narrower with its center shifted down away from the Fermi energy. The shape and position of the Mo d-band DOS do not significantly change, no matter which layer the Mo atom is located at. This indicates that the preference of Mo atoms to be embedded in the Ni bulk is mainly governed by surface energy considerations and atomic size effects. The Mo d-band DOS for the oxygen adsorption cases are largely widened and lead to a strong covalent bonding between the molybdenum atom and the oxygen atom. The present study provides valuable insight for exploring practical applications of Ni-based alloys as hydrogen evolution electrodes.
