# Correction: First-Principles Study of Mo Segregation in MoNi(111): Effects of Chemisorbed Atomic Oxygen. *Materials* 2016, *9*, 5

**DOI:** 10.3390/ma9050352

**Published:** 2016-05-11

**Authors:** Yanlin Yu, Wei Xiao, Jianwei Wang, Ligen Wang

**Affiliations:** 1 General Research Institute for Nonferrous Metals, Beijing 100088, China; yuyanlin_121@163.com (Y.Y.); wxiao@ustb.edu.cn (W.X.); jswjw@sina.com (J.W.); 2 School of Materials Science and Engineering, University of Science and Technology Beijing, Beijing 100083, China; 3 Power Environmental Energy Research Institute, Covina, CA 91722, USA

The authors wish to make the following corrections to this manuscript [1]. The published Figure 1 was incorrect of MoNi(111) alloy systems showing one Mo monomer substituting one Ni atom in the (a) first; (b) second, (c) third; and (d) fourth nickel layer. However, the mistake does not change any calculated results. For the preciseness of academic logic and the right of academic ethics, the authors must point out their mistake, and correct it. The correct Figure 1 is shown below. And the authors are responsible for these errors, they regret any inconvenience or misunderstanding caused by them.

## Figures and Tables

**Figure 1:**
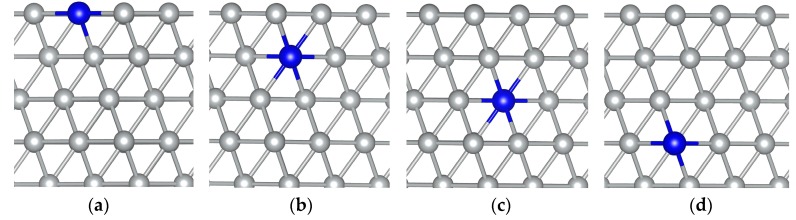
MoNi(111) alloy systems showing one Mo monomer substituting one Ni atom in the (**a**) first; (**b**) second; (**c**) third; and (**d**) fourth nickel layer. Only the four top layers are shown. Gray and blue balls represent Ni and Mo atoms, respectively.
